# Supplementary material for: Tau in cerebrospinal fluid induces neuronal hyperexcitability and alters hippocampal theta oscillations
Source: Acta Neuropathol Commun. 2023 Apr 24;11:67. doi: 10.1186/s40478-023-01562-5 (PMC10127378; doi:10.1186/s40478-023-01562-5)
Supplement: Supplementary file 1 — Additional file 1. Figure 1. Dose response for the effect of CSF-tau on neuronal function. Figure 2. Protocol for analysis of hippocampal CA3 theta oscillations. Figure 3. Validation of tau effects using CSF-mock-depleted. [file 40478_2023_1562_MOESM1_ESM.docx]

**Supplementary material**

**Supplementary figure 1. Dose response for the effect of CSF-tau on neuronal function.** The CSF-tau samples were diluted with artificial CSF (aCSF) to reduce the volume of sample that required for each experiment. As the end goal will be to use individual patient samples (with much smaller volumes ~300 µl) to enable correlation with clinical data, we first sought to find the minimum volume (maximum dilution in aCSF) of CSF-tau, for which we could detect a change in neuronal function. Representative examples of standard current-voltage responses for slices that have been incubated in control aCSF vs different dilutions of CSF in aCSF (1:100, 1:30 and 1:15 dilutions). Neuronal excitability measured using whole-cell patch clamp recording from single CA1 pyramidal cells in the hippocampus was used as a readout. We found no significant changes to neuronal excitability with either the 1:100 dilution or 1:30 dilution of CSF-tau, but a significant depolarisation (**a**; p = 0.0212) and increase in firing rate (**b**; p = 0.0215) was observed with a 1:15 dilution (100 µl CSF in 1.5 ml aCSF) compared to control slices. We then tested this dilution (1:15) using a CSF sample from a healthy 62-year-old female patient, with biomarker-negative amyloid or tau levels. We found that both resting membrane potential and firing rate were not significantly different from the aCSF control group and did not reproduce the changes observed with CSF-tau, with the effects on RMP and FR induced by CSF-tau significantly different to the effects of healthy CSF (RMP p = 0.0120), FR (p = 0.0295).

**
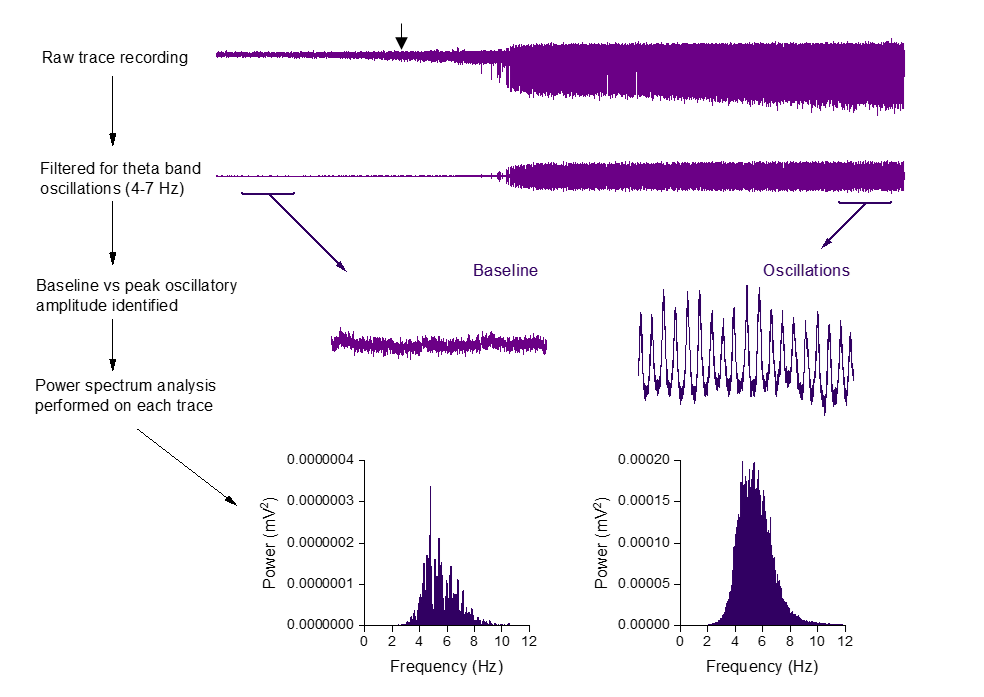
**

**Supplementary figure 2 – Protocol for analysis of hippocampal CA3 theta oscillations.** Carbachol-elicited oscillations were characterised using power spectral density (PSD) analysis in Spike 2. PSD profiles of the field potential recordings filtered for the theta band (4 – 7 Hz) were generated by Fourier transform analysis (Hanning window, FFT size 2048, resolution 4.883 Hz) from each recording. The profiles were calculated from a 100 – 300 second section of the field potential trace displaying peak oscillatory activity, with the baseline power subtracted offline. Power spectrum analysis was performed to ascertain oscillatory power. Arrow denotes start of carbachol application.

**Supplementary figure 3 - Validation of tau effects using CSF-mock-depleted.** To further demonstrate that the observed effects were a result of tau and not an artefact of the immunodepletion protocol, we used an aliquot of the CSF pool, immunodepleted using the same protocol but with antibodies against IgG and neurogranin a. Representative examples of standard current-voltage responses for slices that have been incubated in control aCSF (light blue; n=8) and CSF-mock depleted (purple; n=10). b. Representative example of membrane-potential responses to naturalistic current injection for both conditions. c. CSF-mock-depletion incubation resulted in a significant depolarisation of the resting membrane potential compared to control (aCSF) (p = 0.0018) and compared to CSF-tau-depleted (p = 0.0001) and was not significantly different from incubation with the full CSF-tau sample. d. CSF-mock-depletion incubation resulted in a significant increase in firing rate compared to control (aCSF) (p = 0.0109) and compared to CSF-tau-depleted (p = 0.0481) and was not significantly different from incubation with the full CSF-tau sample. e. Carbachol-induced theta oscillations in the CA3 region of the hippocampus were recorded in an interface chamber (see methods for details). e. Representative examples of theta oscillations for control (aCSF) and CSF-mock-depleted. **f,** mean power spectrums for each of the conditions. CSF-mock-depleted slices had significantly stronger oscillatory power compared to control (aCSF; p = 0.0813) and CSF-tau-depleted (p = 0.0012) and were not significantly different from CSF-tau (full). *Panels a, b and e show representative example traces and c and d show the mean data and SEM, with individual datapoints overlaid.* *Data from control (aSCF) and CSF-mock-depleted were collected in parallel and interleaved). They are presented here in comparison CSF-tau and CSF-tau depleted. The data for these two conditions are replicated from figure 2 and are denoted using a cross pattern.*
